# *Aedes aegypti* abundance, larval indices and risk for dengue virus transmission in Kinondoni district, Tanzania

**DOI:** 10.1186/s41182-021-00395-z

**Published:** 2022-01-04

**Authors:** Baraka L. Ngingo, Leonard E. G. Mboera, Augustino Chengula, Ines Machelle, Mariam R. Makange, Michael Msolla, Gaspary O. Mwanyika, Sima Rugarabamu, Gerald Misinzo

**Affiliations:** 1 Department of Veterinary Microbiology, Parasitology and Biotechnology, Sokoine University of Agriculture, Morogoro, Tanzania; 2 SACIDS Foundation for One Health, Sokoine University of Agriculture, Morogoro, Tanzania; 3 Faculty of Natural and Applied Sciences, St John’s University of Tanzania, Dodoma, Tanzania; 4 Department of Medical Sciences and Technology, Mbeya University of Science and Technology, Mbeya, Tanzania; 5 Department of Microbiology and Immunology, Muhimbili University of Health and Allied Sciences, Dar es Salaam, Tanzania

**Keywords:** Dengue, *Aedes aegypti*, Productivity, Transmission risk, Tanzania

## Abstract

**Background:**

Tanzania has experienced periodic dengue outbreaks with increased incidence since 2010. However, there is limited information on vector dynamics and transmission risk in most parts of the country. This study was conducted to determine *Aedes* mosquito abundance, larval indices and dengue virus infection rate as risk indicators for DENV transmission in Kinondoni district, Dar es Salaam, Tanzania.

**Methods:**

A cross-sectional study was conducted in three wards of Kinondoni district in Tanzania between December 2019 and January 2020. In each ward, three streets were randomly selected for adult and immature mosquito sampling. The adult mosquitoes were collected using Mosquito Magnet traps, while mosquito larvae and pupae were inspected in water-holding containers in the selected household compounds. The detection of dengue virus (DENV) in female *Aedes* mosquitoes was done using a one-step reverse transcription–polymerase chain reaction (RT–PCR) method.

**Results:**

Of the 1416 adult female mosquitoes collected, *Ae. aegypti* accounted for 16.8% (*n* = 238). A total of 333 water-holding containers were inspected and 201 (60.4%) had at least an *Aedes* larvae or pupae. Water-holding containers supporting the breeding of *Aedes* larvae and pupae included discarded car tires, flowerpots and small and large plastic containers. The overall House Index, Container Index and Breteau Index were 55.1%, 60.4% and 114.2, respectively. None of the 763 female *Aedes* mosquitoes tested by RT–PCR was found to be infected with DENV.

**Conclusion:**

The presence and abundance *Ae. aegypti* mosquitoes and the large proportion of water-holding containers infested with the mosquito larvae and pupae put residents of Kinondoni district at high risk of DENV transmission. Our findings emphasize the need for continuous mosquito vector surveillance and control to prevent the possibility of future DENV outbreaks in Tanzania.

## Background

Dengue is a mosquito-borne viral disease of global health concern. The disease has spread throughout the tropical and sub-tropical regions over the past 60 years and currently affects over half of the world’s population [1]. The disease is caused by dengue virus (DENV), an RNA virus with four distinct serotypes, DENV1–4, each capable of causing disease, ranging from mild fever to severe disease [2]. The spread of DENV infection is driven by increased international travel, climate change effect associated with high humidity and temperature, and poor urban environmental conditions that favour mosquito survival, breeding, and abundance [3].

In Tanzania, dengue was first reported in the southern coastal district during the fifteenth Century by Spanish sailors [4–6]. Dengue outbreaks were later reported between 1823 and 1870 on the Islands of Zanzibar archipelago [7]. During the past 10 years, dengue outbreaks in Tanzania have been reported with increased incidence [8–10]. The most recent outbreak in 2019 affected mainly Dar es Salaam and Tanga cities, where a total of 6,917 confirmed cases and 13 deaths (case fatality rate of 0.19%) were reported [11]. Moreover, seroprevalence studies have reported dengue in several regions of Tanzania with rates between 6.4% and 50.6% [8, 10, 12–16]. All the four DENV serotypes, DENV-1, DENV-2, DENV-3 and DENV-4 have been reported in Tanzania [8, 10, 17, 18]. Despite of frequent dengue outbreaks in the country, no vector control measures have been introduced.

Dengue virus is transmitted between humans through a bite of an infected *Aedes* mosquito. The main vectors that are geographically widespread include *Ae. aegypti* and *Ae. albopictus* [2]. *Ae. aegypti* is an extensive domestic day-biting species that prefer to feed on humans. It breeds in flower vases, uncovered barrels, buckets, discarded cans, roof gutters and discarded tires [8, 19] while *Ae. albopictus,* preferentially rests outdoors [20] and alternatively feeds on humans and animals, though it has been reported to exhibit strong anthropophagic behaviour in some countries [21]. In Tanzania, *Ae. aegypti* is the main vector of Dengue [8], no report on presence of *Ae. albopictus*. DENV infection rate of 2% [22] 8% [8] in *Ae. aegypt*i mosquitoes have been reported in Dar es Salaam. Relatively higher DENV infection rate of 47.6% in *Ae. aegypti* have been recorded in a study in Kagera, Mwanza, Morogoro and Mbeya regions [23]. Nevertheless, the extents of viral infections in vector mosquitoes in other parts of the country have not been well established. Screening for DENV serotypes in mosquitoes will give baseline data for future vector control intervention studies.

Though several dengue outbreaks have been reported in Tanzania during the past decade, information on mosquito vectors and transmission indices are limited. So far, five entomological surveys have been reported; of which only three have established transmission levels of DENV [8, 22, 23]. This study was, therefore, carried out to determine *Aedes* mosquito abundance, larval indices and dengue virus infection rate as risk indicators for DENV transmission in Kinondoni district, Dar es Salaam, Tanzania.

## Methods

### Study area and design

This cross-sectional entomological study was carried out in Kinondoni district of Dar es Salaam City in eastern Tanzania between December 2019 and January 2020. The district has a total area of 531 km^2^ with a population of about 1,775,049 and a population density of 3343 inhabitants per km^2^ [24]. The climate is generally hot and humid, with small seasonal and daily variations in temperature. The mean annual temperature and the annual rainfall are about 30 °C and 1100 mm, respectively. The relative humidity is generally high and ranges between 65 and 96% in a year [25]. Kinondoni district was selected purposely, because the district has experienced periodic dengue outbreaks since 2014 [8, 10, 16].

**Fig. 1:**
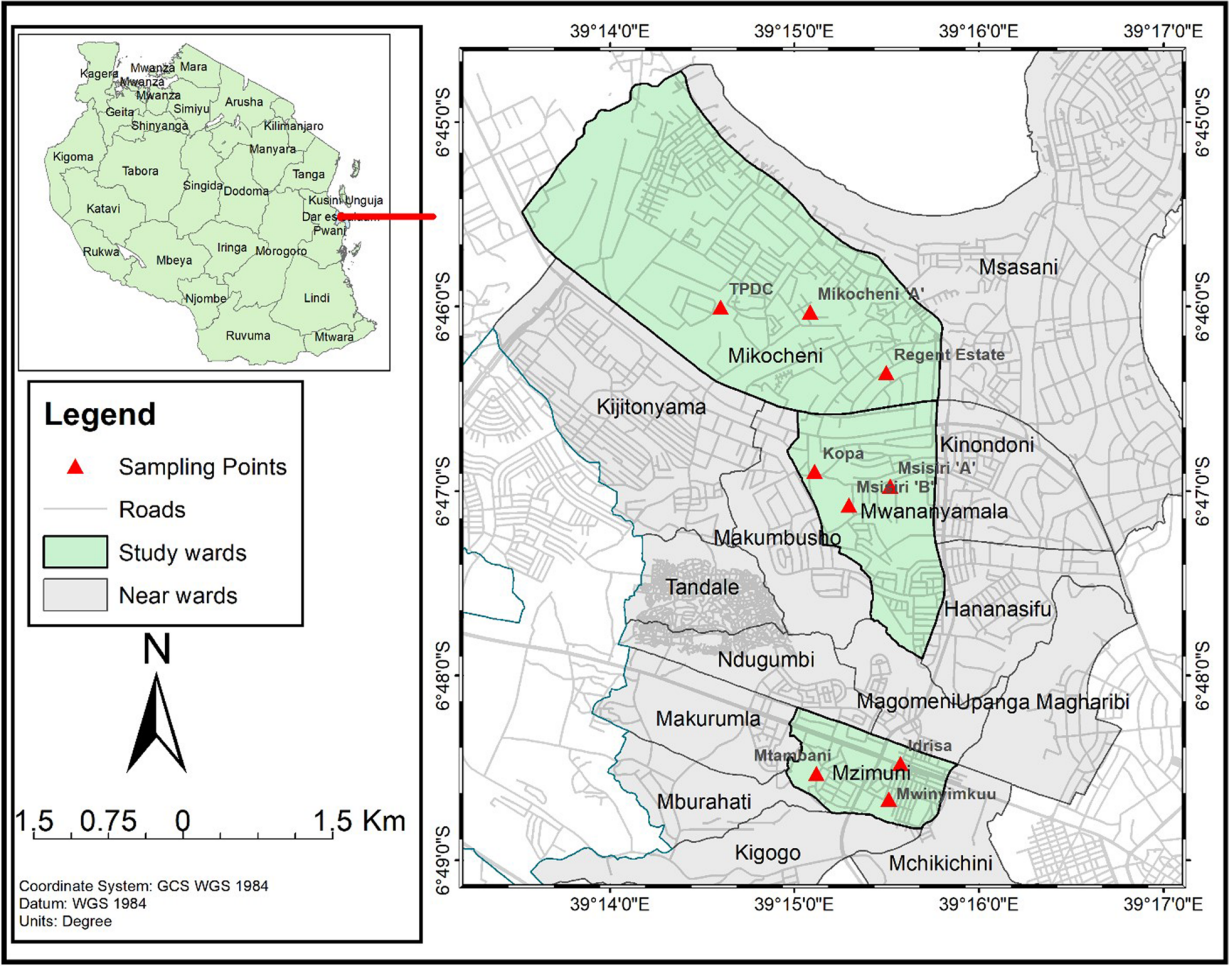
Map of Kinondoni district showing the study wards and sampling sites (in red triangles). The map was developed using ArcGIS software version 10.5 (Esri, CA, USA)

Mosquito sampling was conducted in Mikocheni, Mzimuni, and Mwananyamala wards selected based on ecological and demographical characteristics. Mikocheni is a high-income area characterized by sparsely populated neighbourhood (low population density). Sampling streets have regular blocks with high standard dwellings, high vegetation coverage, piped water supply and regular garbage collection. The houses generally have large and shaded peridomestic environments, while Mwananyamala is a low-income area characterized by unplanned urbanization and high population density. Sampling streets were characterized by low vegetation coverage, insufficient piped water supply and irregular waste collection. Mzimuni is a low to middle-income area characterized by unplanned urbanization and high population density. Sampling streets were characterized by low vegetation coverage, insufficient piped water supply and irregular waste collection. In each ward, three streets were randomly selected as sampling points making a total of nine study streets, namely, Regent Estate, Mikocheni A, TPDC, Idrisa, Mwinyimkuu, Mtambani, Msisiri A, Msisiri B and Kopa (Fig. 1).

### Mosquito sampling and breeding habitat assessment

This study involved sampling of both adult and immature stages of mosquitoes. The sampling procedure used for collecting adult mosquitoes is as described by Mboera et al. [8]. Adult mosquitoes were collected using carbon dioxide–propane powered Mosquito Magnet Liberty Plus traps (American Biophysics Corporation, Rhode Island, USA), fixed at nine sentinel sites (three sites in each sampling ward). A total of nine traps were set, three in each of the three study wards of Kinondoni district and allowed to operate for 3 days consecutively. The traps were set in the morning and operated throughout the day and night. The mosquito catches were collected the following day between 6:00 a.m. and 08:00 a.m., identified morphologically and stored for further analysis. A manual aspirator was used to correct the mosquitoes from the traps [26].

In all streets visited, there was a register book with house number and names of the household owner. All house numbers were taken and sampling frame were prepared in MS Excel. Random numbers were generated for selection of houses for this study. The number of households to be involved was determined as described previously [27]. The operational guide for assessing the productivity of *Ae. aegypti* breeding sites was according to the guidelines by the World Health Organization [28].Twenty randomly selected households in each of the study streets were visited and inspected for the presence of water-holding containers [27]. All areas surrounding the selected household compound were visited and inspected for the presence of water holding containers. The larval search team consisted of five research assistants all visiting and inspecting one house after the other. This was to make sure that all potential water holding containers are thoroughly searched for presence of immature stages of mosquitoes. All water-holding containers found were examined for the presence of mosquito larvae and pupae. A container found with a larva or pupa was considered positive for the presence of immature mosquito stages. Larvae and pupae found were collected into a small bowl using standard plastic dippers and transferred using Pasteur pipette into a labelled water-filled Whirl–Pak plastic bag (Thomas Scientific, Swedesboro, USA). The collected larvae and pupae were taken in a cool box to an insectary at the Muhimbili University of Health and Allied Sciences in Dar es Salaam for rearing. In the insectary, the larvae were fed on Whiskas® cat food and maintained at 26 ± 2 °C and relative humidity of 82 ± 10%. Pupae were collected by trained staff each morning and evening and transferred to pupae emergence cups in netting cages and left until adults emerged. Upon emergence, adult mosquitoes were euthanized at −20 °C for 15 min and identified morphologically to the species level with the aid of a stereo-microscope [26].

### Mosquito processing

Twenty females *Ae. aegypti* were pooled into well-labelled 1.5 mL cryovial tubes according to the collection site and whether captured or reared and then frozen in liquid nitrogen (−196 °C). The mosquito specimens were transported to the Sokoine University of Agriculture Laboratory in Morogoro, Tanzania. In the laboratory, the pooled specimens were placed in a 1.5 mL microcentrifuge tube with 500 µL of Dulbecco’s Modified Eagle Medium (Sigma-Aldrich Chemie GmbH, Taufkirchen, Germany), ground and centrifuged at 12,000 rpm for 1 min and 200 µL of the supernatant was stored at −80 °C until analysed.

### Screening of DENV

Viral ribonucleic acid (RNA) was extracted from each pool of mosquitoes using QIAamp Viral RNA Kit (Qiagen, Hilden, Germany), according to the manufacturer’s instructions. RNA was eluted in 60 µL of elution buffer provided with the kit. The eluted RNA was aliquoted into three aliquots of 20 µL each and immediately stored at -20 ºC until analysed. The quality and quantity of RNA was evaluated using the NanoDrop ND1000 spectrophotometer (Biochrom LTD, Cambridge, England). One-step reverse transcription–polymerase chain reaction (RT–PCR) was done using SuperScript III Platinum/Taq DNA polymerase kit (Invitrogen, Carlsbad, CA, USA) according to the manufacturer’s instructions using D1 and D2 primers that amplify a 511 bp fragment of the polyprotein gene at 134 and 644 genomic position [29].

Reverse transcription–polymerase chain reaction was carried out in a 25 µL reaction containing 12.5 µL of 2 × reaction mix, 1 µL of SuperScript III RT/Platinum Taq mix, 0.5 µL of 10 μM sense primer (D1), 0.5 µL of 10 μM anti-sense primer (D2), 0.5 µL Magnesium sulphate (Invitrogen, CA, USA), 4 µL of RNA template and 6 µL of nuclease-free water. Reverse-transcription reaction was performed in 1 cycle at 48 ºC for 30 min, followed by one cycle of initial denaturation at 94 ºC for 2 min. Thermocycling conditions were 35 cycles of denaturation at 94 ºC for 15 s, annealing at 55 ºC for 30 s, and elongation at 68 ºC for 1 min and a final extension at 68 ºC for 5 min. The PCR amplicons were separated on 1.5% agarose gel electrophoresis and visualized using Gel doc EZ Imager System (Bio-Rad Laboratories, Hercules, CA, USA).

### Data analysis

Data were entered into Microsoft Excel sheet 2016 (Microsoft Corporation, Washington, USA), cleaned and evaluated against data collection forms. The proportion of mosquitoes by species and wards, households with water holding container were calculated and presented in tables. Risk for DENV transmission was assessed using dengue risk indicators, including *Aedes* mosquito abundance, larval indices and dengue virus infection rate. The level of *Aedes* mosquito infestation and dengue transmission levels were assessed by: (i) House index as the percentage of houses infested with larvae and/or pupae; (ii) Container index as the percentage of water-holding containers infested with larvae and/or pupae; and (iii) Breteau index as the number of positive containers per 100 houses inspected in a specific location. From these indices, potential risk of dengue transmission was estimated based on the World Health Organization criteria, which states that where the BI, HI and CI exceeded 50, 35 and 20, respectively, the risk of dengue transmission is considered high. Where the BI is between 5 and 50, the density of *Ae. aegypti* was considered sufficient to promote an outbreak of Dengue. Where the BI was less than 5, the HI less than 4, and the CI was also less than 3, it is considered unlikely for transmission of dengue to occur [30].

Chi-square and *t* test were used to test for significant differences in indices and mosquito female *Ae. aegypti* abundance between study wards, respectively. All the results with p < 0.05 were considered statistically significant.

## Results

### Mosquito species composition and abundance

A total of 1,416 adult female mosquitoes were collected outdoors using Mosquito Magnet traps. *Culex quinquefasciatus* was the most abundant mosquito species accounting for 52.3% (*n* = 740) of the total female mosquitoes collected. *Mansonia* species and *Ae. aegypti* accounted for 30.9% (*n* = 438) and 16.8% (*n* = 238), respectively. A statistically significant variation in female *Ae. aegypti* abundance between study wards was observed (*p* < 0.05). The largest proportion of *Ae. aegypti* mosquitoes were collected from Mikocheni (Table 1). A total of 1,750 *Ae. aegypti* mosquitoes hatched from the larvae and pupae reared in the insectary (Table 2). 30% of the female *Ae. aegypti* mosquitoes hatched in the respective sampling ward were randomly selected and packed for detection of DENV virus.

**Table 1:** Number and percentage of adult mosquitoes collected by ward and species

Ward	Species	No	Percentage
Mikocheni	*Aedes aegypti*	148	42.4
	*Culex quinquefasciatus*	201	57.6
	Subtotal	349	24.6
Mwananyamala	*Aedes aegypti*	36	4.0
	*Culex quinquefasciatus*	436	47.9
	Mansonia spp.	438	48.1
	Subtotal	910	64.3
Mzimuni	*Aedes aegypti*	54	34.4
	*Culex quinquefasciatus* Subtotal	103 157	65.6 11.1
	Total	1416

**Table 2:** Number of hatched mosquitoes by sampling location

Ward	Total *Ae. aegypti* corrected	No of *Ae. aegypti* screened for DENV
Mikocheni	876	263
Mwananyamala	328	98
Mzimuni	546	164
Total	1750	525

### Mosquito breeding sites and Aedes indices

A total of 176 houses were inspected for the presence of immature stages of *Aedes* mosquito. Of these, 132 (75%) houses had water-holding containers in their premises. Mikocheni had the largest proportion (85%) of households with water-holding containers, followed by Mzimuni (75.9%) and Mwananyamala (63.8%). About two-thirds (60.4%) of the household premises had at least a container with mosquito larvae or pupae. A total of 333 outdoor containers were found and inspected for the presence of *Ae. aegypti* larvae or pupae (Table 3). Of 109 discarded car tires inspected, 94 (80.2%) were infested with *Ae. aegypti* immature stages. Other water holding containers infested with *Aedes* larvae/pupae were small plastic containers, flower pots, water buckets, unused fish pond and water drums (Table 3). Discarded car tires accounted for the largest proportion (76%) of water-holding containers harbouring pupae. About two-thirds (60%) of the flower pots and cement water tanks were also infested with pupae (Table 3).

**Table 3:** Type and number of water-holding containers infested with mosquito larvae or pupae

Type of container	Number of containers (containers infested with pupae)							
	**Mikocheni**		**Mwananyamala**		**Mzimuni**		**Total**	
	**Larvae**	**Pupae**	**Larvae**	**Pupae**	**Larvae**	**Pupae**	**Larvae**	**Pupae**
Cement water tank (1000 L)	3 (2)	3 (1)	1 (1)	1 (1)	1 (1)	1 (1)	5 (4)	5 (4)
Flower pot	11 (7)	11 (3)	4 (3)	4 (3)	6 (6)	6 (6)	21 (16)	21 (12)
Plastic container (5–20 L)	5 (3)	5 (2)	2 (0)	2 (0)	10 (3)	10 (2)	17 (6)	17 (4)
Plastic water drum (200 L)	3 (2)	3 (1)	4 (3)	4 (3)	3 (1)	3 (1)	10 (6)	10 (5)
Water tank (> 200 L)	7 (7)	7 (5)	4 (2)	4 (2)	4 (0)	4 (0)	15 (9)	15 (7)
Discarded car tires	45 (38)	45 (27)	19 (18)	19 (18)	45 (38)	45 (38)	109 (94)	109 (83)
Plastic container (< 5 L)	4 (4)	4 (1)	5 (4)	5 (4)	6 (4)	6 (3)	15 (12)	15 (8)
Water bucket (20 L)	19 (8)	19 (5)	26 (12)	26 (9)	40 (5)	40 (3)	85 (25)	85 (17)
Water bottle (12 L)	9 (4)	9 (4)	5 (0)	5 (0)	2 (0)	2 (0)	16 (4)	16 (4)
Others	17 (15)	17 (7)	11 (4)	11 (3)	12 (7)	12 (7)	40 (26)	40 (17)
Total	123 (90)	123 (56)	81 (46)	81 (43)	129 (65)	129 (61)	333 (201)	333 (160)

**Fig. 2:**
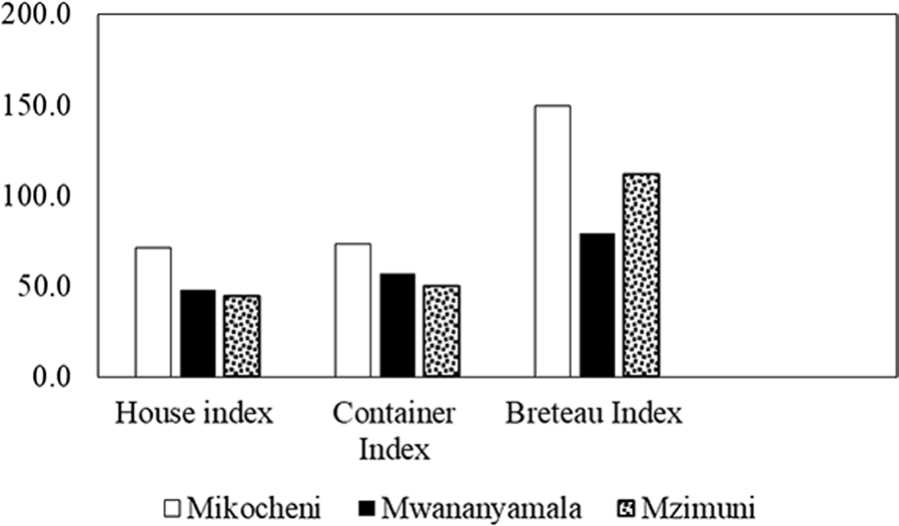
House, Container and Breteau indices by ward

The overall *Aedes* house index (HI) was 55.1%. The highest HI (71.7%) was found in Mikocheni, followed by Mwananyamala (48.3%) and Mzimuni (44.8%). There was a significance difference in House indices between wards (*x̄* = 54.93, 95% CI: 18.6–91.2 ± 14.6, *p* = 0.023). The overall container index (CI) was 60.4%, with Mikocheni having significantly the higher CI (73.2%) than Mwananyamala (56.8%) and Mzimuni (50.4%) (*x̄* = 60.13, 95% CI: 30.9–89.3 ± 11.8, *p* = 0.013). The overall Breteau index was 114.2, with the highest value found in Mikocheni (150), followed by Mzimuni (112.1) and Mwananyamala (79.3). The difference in Breteau indices between wards was statistically significant (*x̄* = 113.8, 95% CI: 25.9–202.0 ± 35.4, *p* = 0.031) (Fig. 2).

### Dengue virus detection

**Fig. 3:**
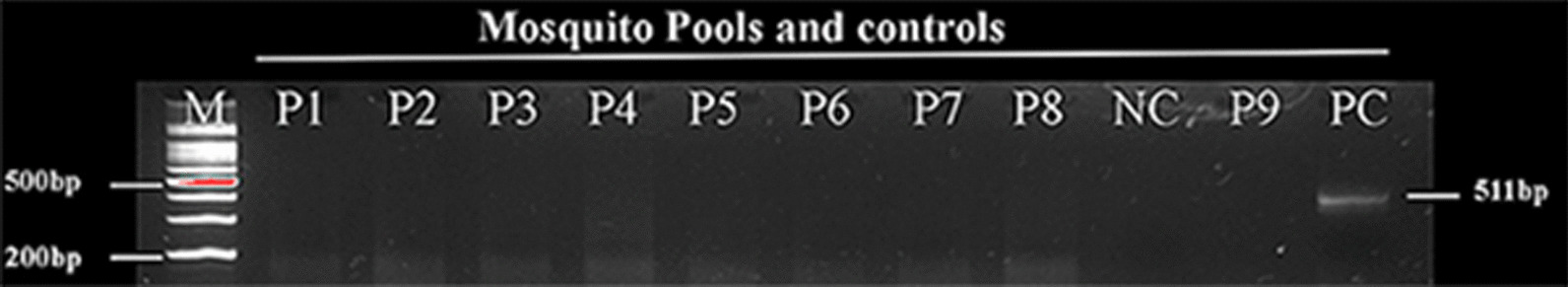
Agarose gel electrophoresis image of RT–PCR test results for dengue virus in female *Ae. aegypti* mosquitoes. M is a 100 bp DNA ladder, P1–9 are representative of mosquito pools tested (samples), PC—positive control and NC—negative control

Overall, 51 pools consisting of 763 (238 trapped and 525 hatched) females *Ae. aegypti* were tested for DENV infection using one-step RT–PCR. Of these, 22 pools were from field trapped adult mosquitoes and 29 pools were from emerged pupae. All the mosquito pools tested negative for DENV (Fig. 3).

## Discussion

Although dengue virus was not detected from mosquitoes, *Aedes* mosquito abundance and larvae indices were high in Kinondoni district. *Ae. aegypti* accounted for less than one-fourth of the adult female mosquitoes collected. The majority of *Ae. aegypti* were collected from Mikocheni ward. Similar findings were previously reported in Dar es Salaam [8, 31]. In comparison, Mboera et al. found five mosquito species in the same district in a study conducted during the dengue outbreak of 2014/2015 [8]. The study by Mboera et al. was conducted during the long rainy season (April–May) [8], compared with this study that was carried out during the end of a short rainy season (December) and the beginning of the dry season (January). The effect of seasonal variations on the abundance and distribution of *Aedes* mosquitoes has been reported by other authors [32, 33].

Results from house survey show that three-quarters of the houses in Kinondoni had water-holding containers that were potential breeding sites of *Aedes* mosquitoes. More than half of the inspected houses had water-holding containers infested with *Aedes* mosquito larvae and or pupae. The overall house index in our study was lower than that previously reported in the same district [8], most likely due to the seasonal differences. Results from a study in northern Ghana also showed higher house indices ranging from 55.9% to 88.3% [34]. The overall container index in the current study was higher than previously reported [8]. The findings from another study in the rural Ifakara district of south-eastern Tanzania revealed relatively lower larvae indices than those found in this study [35]. High *Aedes* mosquito indices reported in this study were not surprising as the study was carried out during the end of the short rainy and the start of the dry season [25]. The high Breteau index reported in this study indicates that the residents of Kinondoni are likely to be at high risk of dengue virus transmission [36]. These findings agree with the results reported from a study in northern Ghana, where Breteau indices of 72.4 to 180.9 were reported during a dry season [34]. The higher larval indices observed in Mikocheni compared to the other wards could be explained by several factors, including fact that Mikocheni is high-income ward characterized by houses with large and shaded peridomestic environments (gardens) with potted flowers, which are potential mosquito breeding habitats. These observations call for the need to improve *Aedes* mosquito control practices in the study district to reduce the risk of DENV transmission.

In the house survey, it was found that the most common breeding containers for the *Aedes* mosquitoes were discarded car tires and flower pots. It is a presumption that a large number of water-holding containers with immature mosquito stages found outdoors in Kinondoni could be a reason for the abundance of *Ae. aegypti* reported in this study. Similarly, a previous study found that water-holding containers left outdoors were harbouring *Aedes* immature stages in Kinondoni and other districts of Dar es Salaam [8]. *Ae. aegypti* has also been reported as a common outdoor breeding mosquito in the rural areas of Tanzania [35]. The shortage of water supply may have led to many households keeping containers for water storage in their houses. Furthermore, the flower pots that retain water and improper disposal of used tires are likely to provide suitable sites for *Aedes* mosquito breeding. These factors probably contributed to the high number of breeding sites that favoured high *Aedes* abundance [37]. Highly pupae productive containers were observed to be discarded car tires, cement water tanks and flower pots. Although all the water-holding containers were found to have pupae, mosquito control measures targeting breeding habitats should be directed to water-holding containers perceived to hold water for longer periods. Similar observations were reported from other studies in Dar es Salaam, Tanzania [8, 38] and Mombasa Kenya [35, 36].

Laboratory analysis showed that DENV was not detected in the mosquito pools tested. In contrary, a previous study in Dar es Salaam reported an infection rate of 6.8% [8]. The absence of DENV infection in *Aedes* mosquitoes has also been reported in a study in Kenya [39]. The absence of infection could be due to several factors, including lower average precipitation [40], number of mosquitoes tested, high ratio of hatched mosquitoes compared to the total number of mosquitoes tested and the absence of DENV circulating in humans during the study period [39]. Although DENV was not detected in *Aedes* mosquitoes, their abundance and the large proportion of water-holding containers infested indicate the risk of DENV transmission in the study areas is high. A recent study reported Kinondoni district to have the highest seroprevalence of dengue than elsewhere in Tanzania [16].

## Conclusion

This study reveals the presence and abundant *Ae. aegypti* mosquitoes and a large proportion of water-holding containers infested with larvae and pupae in Kinondoni district, Tanzania, suggesting that residents of the district are at higher risk of DENV transmission.Although DENV was not detected, our findings emphasize the need for continuous mosquito vector surveillance in Tanzania to guide strategies for appropriate vector control to prevent the possibility of future DENV outbreaks.

## Limitation of the study

The few numbers of adult, trap collected female *Ae. aegypti* mosquitoes observed in this study may be due to sampling period of the year which is the start of dry season. In this study, the virus detection rate is also considered to be low because of the high ratio of hatched mosquitoes compared to the total number of mosquitoes tested. This was a cross-sectional study and hence its findings cannot be used to interpret the seasonal abundance of the mosquito.

## Data Availability

The data sets used and/or analysed during the current study are available from the corresponding author upon request.

## Abbreviations

DENV: Dengue virus
RT-PCR: Reverse transcription-polymerase chain reaction
CI: Container index
HI: House index
BI: Breteau index
